# Coronary artery disease risk in young women with polycystic ovary syndrome

**DOI:** 10.18632/oncotarget.23985

**Published:** 2018-01-04

**Authors:** Dah-Ching Ding, I-Ju Tsai, Jen-Hung Wang, Shinn-Zong Lin, Fung-Chang Sung

**Affiliations:** ^1^ Department of Obstetrics and Gynecology, Hualien Tzu Chi Hospital, Buddhist Tzu Chi Medical Foundation, Tzu Chi University, Hualien, Taiwan; ^2^ Institute of Medical Sciences, Tzu Chi University, Hualien, Taiwan; ^3^ Management Office for Health Data, China Medical University Hospital, Taichung, Taiwan; ^4^ College of Medicine, China Medical University, Taichung, Taiwan; ^5^ Department of Research, Hualien Tzu Chi Hospital, Buddhist Tzu Chi Medical Foundation, Tzu Chi University, Hualien, Taiwan; ^6^ Department of Neurosurgery, Hualien Tzu Chi Hospital, Buddhist Tzu Chi Medical Foundation, Tzu Chi University, Hualien, Taiwan; ^7^ Department of Health Services Administration, China Medical University, Taichung, Taiwan

**Keywords:** cardiovascular disease, coronary artery disease, diabetes mellitus, hypertension, polycystic ovary syndrome

## Abstract

Women with polycystic ovary syndrome are characterized by obesity, menstruation irregularity, hirsutism and infertility, and prevalent with cardiometabolic comorbidities, but population-based studies on the risk of developing coronary artery disease are limited. From claims data of the Taiwan National Health Insurance, we identified 8048 women with polycystic ovary syndrome aged 15-49 years newly diagnosed in 1998-2013, and 32192 women without the syndrome and CAD as controls, frequency matched by age and diagnosis date. By the end of 2013, after a mean follow-up period of 5.9 years, the overall incidence of coronary artery disease was 63% higher in women with polycystic ovary syndrome than in controls (2.25 vs. 1.38 per 1000 person-years). The adjusted hazard ratio [aHR] of coronary artery disease was 1.44 (95% confidence interval (CI) = 1.14–1.81) for women with polycystic ovary syndrome, compared with controls. Hazards of coronary artery disease were significant during follow-up periods of 3-4 years (aHR = 1.52, 95% CI = 1.00–2.30) and of 5–9 years (aHR = 1.58, 95% CI = 1.07–2.32). The incidence of coronary artery disease increased further in those with cardiometabolic comorbidities. Among women with polycystic ovary syndrome, those with comorbid diabetes had an incidence of 35.2 per 1000 person-years, 20-fold greater than those without cardiometabolic comorbidities. In conclusion, women with polycystic ovary syndrome are at an elevated risk of coronary artery disease. Preventive interventions should be provided to them, particularly for those with the comorbidity of metabolism symptom.

## INTRODUCTION

Polycystic ovary syndrome (PCOS) is a common endocrine disorder in women of reproductive ages. It may affect 5–10% of women [1]. Women with PCOS may present features of hyperandrogenism, menstrual irregularity and a polycystic morphology of the ovary [2]. PCOS is one of infertility causes due to anovulation [3]. PCOS patients are prevalent with insulin resistance and insulin secretory defects and androgen excess [4–6]. They are also correlated with multiple comorbidities, such as obesity, coronary artery disease (CAD) and cancer [6]. The prevalence of coronary artery calcification (CAC) is 4-fold higher in PCOS patients than in general community women (39.0% vs. 9.9%) [7]. The Coronary Artery Risk Development in Young Adults (CARDIA) study also reported recently that PCOS patients are prevalent with CAC with an adjusted odds ratio of 2.70 [8]. These women are thus at an increased risk of development of subclinical CAD [7]. The hazards of developing coronary heart disease could increase to 12.4-fold for younger individuals with CAC >100, compared with individuals without it [9]. Cardiovascular diseases, including CAD, have been the major causes of deaths not only in western population but also in Asian population with a diverse pattern [10–13]. The CAD mortality in Japan is lower than that in western countries, while the mortality in India is over 6-fold greater than that in Japan or 2-fold greater than that in the US [12].

Diabetes mellitus (DM), hypertension, atrial fibrillation (AF) and chronic kidney disease (CKD) are also comorbidities prevalent in patients with CAD [14–16]. PCOS patients may exhibit a cluster of similar metabolic and vascular abnormalities including obesity, dyslipidemia, hypertension and diabetes [17–20]. A systemic study has shown that women with PCOS and women with CAD share similar risk factors in a cluster [20]. Risk factors for cardiovascular diseases are more common in PCOS women than in women without PCOS. PCOS women are more prevalent with coronary plaque and at a greater moetality risk from CAD than women without PCOS. It is likely that women with PCOS are at a higher risk of developing CAD in later life. However, studies on this association were limited and have remained elusive [20–23]. Long-term study evaluating this relationship is needed.

We, therefore, conducted a nationwide population-based retrospective cohort study, using the 16-year claims data of National Health Insurance (NHI) of Taiwan, to evaluate the association between PCOS and the risk of CAD after accounting for traditional risk factors.

## MATERIALS AND METHODS

### Study population and data source

Data used in this study were extracted from the Longitudinal Health Insurance Database 2000 (LHID2000). The LHID2000 consists of registration files and original claims data from 1996 to 2013 of 1 million beneficiaries randomly selected from the National Health Insurance Research Database (NHIRD; details available at http://nhird.nhri.org.tw/en/index.htm). The coding system of International Classification of Diseases, 9th Revision, Clinical Modification (ICD-9-CM) was used to assign disease diagnoses.

### Study cohorts with and without PCOS

**Figure 1:**
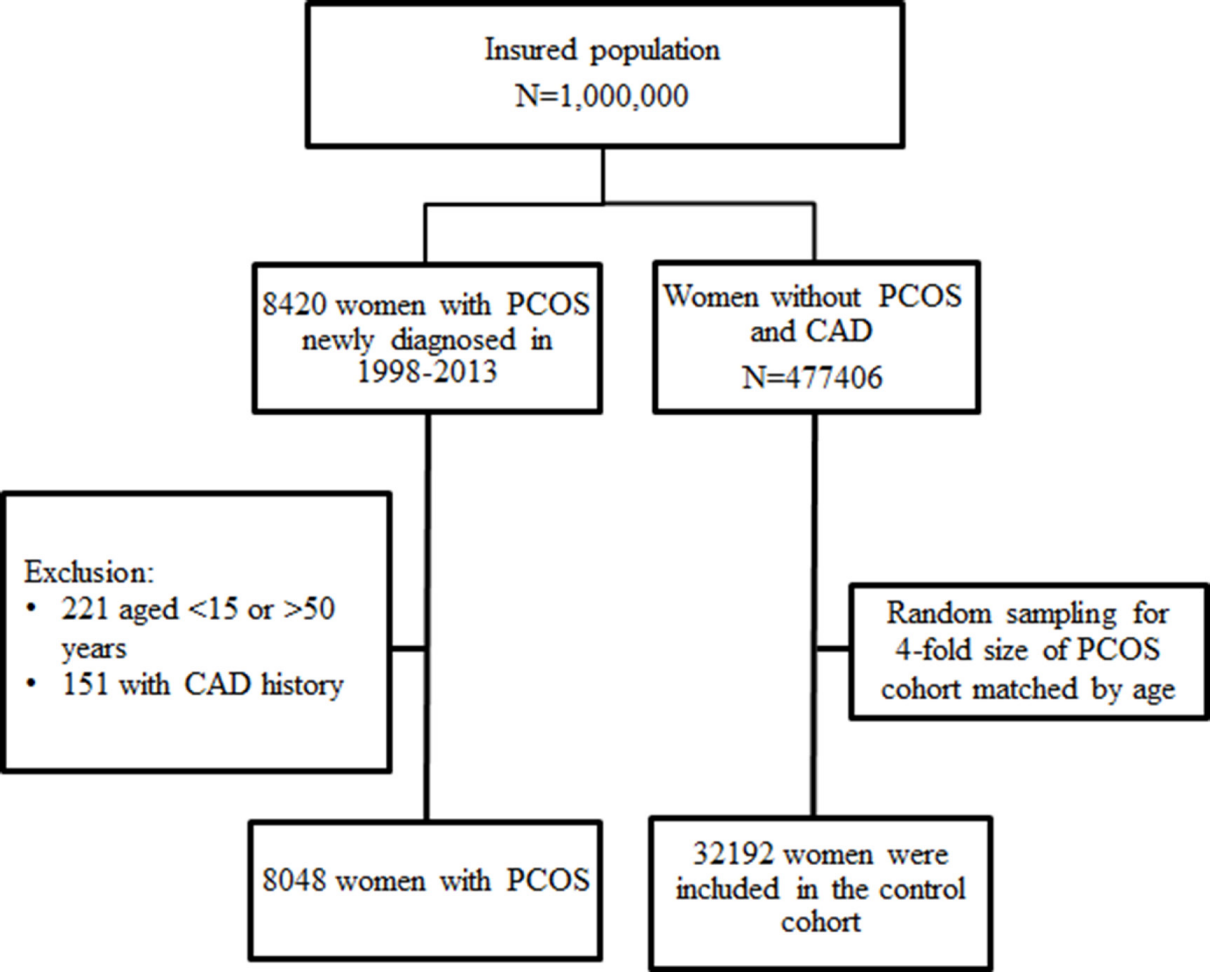
Flow chart for establishing study cohorts with and without polycystic ovary syndrome

Women aged 15-49 years, newly diagnosed with PCOS (ICD9 code: 256.4) between 1998 and 2013, were identified for the potential PCOS cohort. Valid diagnoses were based on blood tests for luteinizing hormone, follicle-stimulating hormone and testosterone (NHI codes: 09078B2, 09126B, 09126C, 09078B1, 09125B, 09125C, 09064B2, 09121B, and 09121C) and/or ultrasonography (NHI code: 19003C). Diseases in this study were identified if there were at least 2 related outpatient diagnoses or at least one related inpatient diagnosis for valid diagnosis. We excluded women with the history of coronary artery disease (CAD) (ICD9 codes: 410-414) before the diagnosis of PCOS. We randomly selected a control cohort from women who were free of CAD at the baseline and never had a PCOS diagnosis, frequency matched by age (exact year) and diagnosis year with the sample size 4-fold of the PCOS cohort. Individuals with missing data of sex, birth date and insurance status were not enrolled in the study cohorts (Figure 1).

Both cohorts were followed until the occurrence of CAD, death, or withdrawal from the NHI program, or December 31, 2013. CAD is a blockage of the major blood vessels (coronary arteries) creating an insufficient supply the heart with blood, oxygen and nutrients because of inflammation and cholesterol plaque in coronary arteries [13].

In this study, baseline comorbidities considered as potential covariates included obesity (ICD9 codes: 278, V77.8, 783.1), hirsutism (ICD9 code: 704.1), irregular menstruation (ICD9 code: 626.4), infertility (ICD9 code: 628), diabetes (DM; ICD9 code: 250), hypertension (HTN; ICD9 codes: 401-405), atrial fibrillation (AF; ICD9 code: 427.31), hyperlipidemia (HL; ICD9 code: 272) and chronic kidney disease (CKD; ICD9 codes: 585, 586, 588.8, 588.9). A disease diagnosis without valid supporting clinical findings may be considered as a medical fraud by the insurance authority with a penalty of 100-fold of the payment claimed by the treating physician or hospital.

### Statistical analysis

The differences in baseline characteristics between PCOS and control cohorts were examined by Chi-square tests for categorical variables and Wilcoxon rank sum test for continuous variables. To estimate the risk of developing CAD, we calculated incidence density of CAD for both cohorts during the follow-up period. The Kaplan-Meier method was used to calculate and plot the cumulative incidence rates, with the difference to be examined using the log-rank test. Cox proportional hazards regression analysis was used to estimate the PCOS cohort to the control cohort hazard ratios (HRs) of CAD with 95% confidence intervals (95% CIs). Incidence and HRs were also evaluated by the follow-up time periods of < 2, 3–4, 5–9, >10 years. Further data analysis pooled both cohorts to evaluate whether the risk of CAD in PCOS women changed in association with disease entities including obesity, irregular menstruation and infertility, compared to women without these factors; similar analysis was performed for the association with comorbidities of diabetes, hypertension and hyperlipidemia, compared to women without these factors. The adjusted HRs were calculated after controlling for age and baseline comorbidities. We also estimated adjusted HRs after controlling for age and lifetime comorbidities.

All statistical analyses were performed using SAS software version 9.4 (SAS Institute Inc., Cary, NC). A two-tailed t-test with -value below 0.05 was considered as significant.

## RESULTS

The study population included 8048 women in the PCOS cohort and 32192 women in the control cohort, with 62.5% of them aged 15-29 years. All disease entities and comorbidities were more prevalent in the PCOS cohort than in the control cohort, particularly for irregular menstruation (66.4% vs.28.0%) and infertility (20.4 vs.3.48%). The mean follow-up periods were similar in the two cohorts (mean ± SD, 5.90 ± 3.98 vs. 5.84 ± 3.96, *p* = 0.24) (Table 1).

**Table 1:** Baseline characteristics compared between polycystic ovary syndrome and control cohort

	PCOS	Controls	*p*-value
	(*N* = 8048)	(*N* = 32192)	
	*n* (%)	*n* (%)	
Age, years			
Mean (SD)	28.11 (6.89)	28.11 (6.90)	0.96^a^
15–29	5026 (62.5)	20159 (62.6)	0.95
30–39	2534 (31.5)	10072 (31.3)	
40–49	488 (6.06)	1961 (6.09)	
Follow-up duration, years			
Mean (SD)	5.90 (3.98)	5.84 (3.96)	0.24^a^
Disease entity			
Obesity	320 (3.98)	303 (0.94)	< 0.001
Hirsutism	64 (0.80)	17 (0.05)	< 0.001
Irregular menstruation	5346 (66.4)	9303 (28.9)	< 0.001
Infertility	1643 (20.4)	1119 (3.48)	< 0.001
Comorbidity			
Diabetes	51 (0.63)	99 (0.31)	< 0.001
Hypertension	237 (2.94)	470 (1.46)	< 0.001
Hyperlipidemia	462 (5.74)	757 (2.35)	< 0.001
Atrial fibrillation	5 (0.06)	9 (0.03)	0.14
Chronic Kidney disease	34 (0.42)	73 (0.23)	0.002
Atherosclerosis	10 (0.12)	19 (0.06)	0.05

PCOS, Polycystic ovary syndrome; SD, standard deviation. Chi-square test was used to examine categorical variable and ^a^Wilcoxon rank sum test was used to examine the continuous variables.

**Figure 2:**
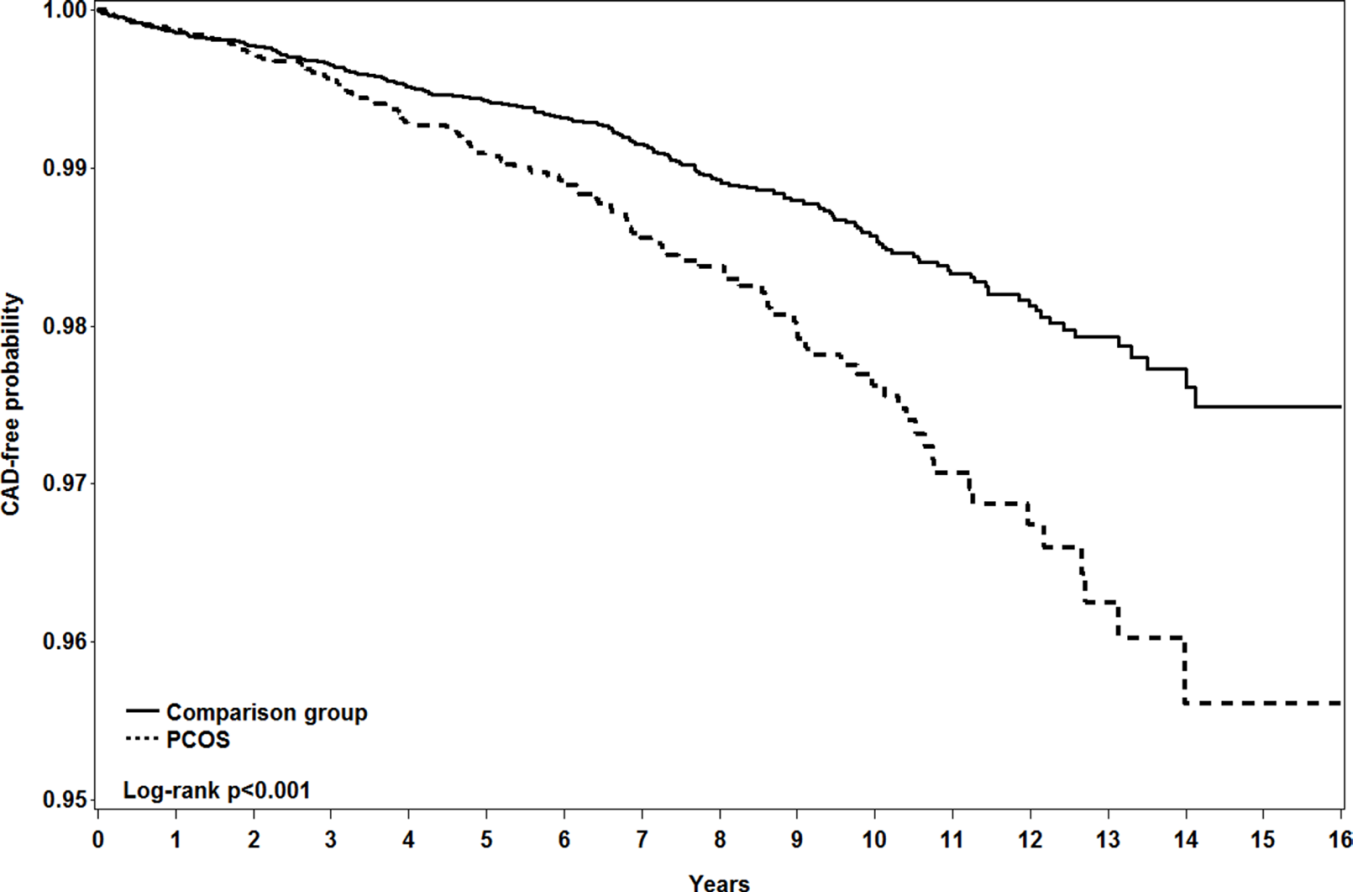
Kaplan-Meier method measured probability free from coronary artery disease in women with (dashed line) and without (solid line) polycystic ovary syndrome

Figure 2 shows the CAD-free probabilities by the end of follow-up for both cohorts. The overall incidence of CAD was 1.95% greater in PCOS women than in controls (Log-rank *p* < 0.001).

The overall incidence of CAD was 1.6-fold greater in PCOS patients than in controls (2.25 vs. 1.38 per 1000 person-years), with an adjusted HR of 1.44 (95% CI = 1.14–1.81) for the PCOS cohort after controlling for age and comorbidities (Table 2). The significant risk of developing CAD in the PCOS cohort appeared during the follow-up years of 3–4 and 5–-9, with adjusted HRs of 1.52 (95% CI = 1.00–2.30) and 1.58 (95% CI = 1.07–2.32), respectively. During follow-up years of 10–16, the incidence of CAD was near 2-fold greater in the PSCO cohort than in the controls, but the adjusted HR of CAD for PSCO women was not significant.

**Table 2:** Incidence and hazard ratio of coronary artery disease in women with polycystic ovary syndrome compared with control cohort by follow-up time

	N	Event	Person-years	Incidence^*^	Hazard ratio (95% CI)	
					Crude	Adjusted
Overall						
Controls	32192	259	188070	1.38	1 (reference)	1 (reference)
PCOS	8048	107	47495	2.25	**1.63 (1.30, 2.04)**	**1.44 (1.14, 1.81)**
Follow-up duration, years						
< 2						
Controls	32192	66	57603	1.15	1 (reference)	1 (reference)
PCOS	8048	20	14445	1.38	1.21 (0.73, 1.99)	0.98 (0.59, 1.65)
3–5						
Controls	25421	75	63396	1.18	1 (reference)	1 (reference)
PCOS	6405	34	15996	2.13	**1.80 (1.20, 2.69)**	**1.52 (1.00, 2.30)**
5–9						
Controls	16927	89	54622	1.63	1 (reference)	1 (reference)
PCOS	4282	38	13822	2.75	**1.69 (1.15, 2.47)**	**1.58 (1.07, 2.32)**
≥ 10						
Controls	5594	29	12449	2.33	1 (reference)	1 (reference)
PCOS	1428	15	3233	4.64	**1.99 (1.07, 3.72)**	**1.87 (0.99, 3.55)**

^*^per 1000 person-years; CI, confidence interval; PCOS, polycystic ovary syndrome The adjusted hazard ratios were measured using Cox Model after controlling for age, diabetes, hypertension, hyperlipidemia, atrial fibrillation, chronic kidney disease and atherosclerosis.

For PCOS women with obesity, irregular menstruation and infertility presented together, the incidence of CAD increased to 13.4 per 1000 person-years with an adjusted HR of 4.90 (95% CI = 1.53–15.7) compared to non-PCOS women free of these three characteristics (Table 3).

**Table 3:** Incidence and hazard ratio of coronary artery disease associated with polycystic ovary syndrome, obesity, irregular menstruation and infertility

PCOS	Obesity	Irregular menstruation	Infertility	N	Event	Person-years	Incidence^*^	Hazard ratio (95% CI)	
								Crude	Adjusted
N	N	N	N	22366	192	139999	1.37	1 (reference)	1 (reference)
N	Y	N	N	167	3	772	3.89	3.03 (0.97, 9.48)	1.01 (0.32, 3.23)
N	N	Y	N	8421	50	41459	1.21	0.95 (0.70, 1.31)	0.94 (0.68, 1.28)
N	N	N	Y	351	4	1915	2.09	1.58 (0.59, 4.25)	1.15 (0.43, 3.09)
N	Y	Y	N	119	2	484	4.13	3.41 (0.85, 13.7)	1.08 (0.26, 4.44)
N	N	Y	Y	751	8	3360	2.38	1.92 (0.95, 3.90)	1.55 (0.76, 3.15)
N	Y	N	Y	5	0	28	0	-	-
N	Y	Y	Y	12	0	52	0	-	-
Y	N	N	N	2163	34	14588	2.33	**1.66 (1.15, 2.39)**	**1.58 (1.09, 2.28)**
Y	Y	N	N	98	3	641	4.68	**3.54 (1.13, 11.1)**	1.43 (0.45, 4.59)
Y	N	Y	N	3951	39	21693	1.8	1.37 (0.97, 1.93)	1.35 (0.96, 1.92)
Y	N	N	Y	417	10	2804	3.57	**2.49 (1.32, 4.71)**	1.82 (0.96, 3.46)
Y	Y	Y	N	173	0	946	0	-	-
Y	N	Y	Y	1177	13	6461	2.01	1.54 (0.88, 2.70)	1.30 (0.74, 2.28)
Y	Y	N	Y	4	0	28	0	-	-
Y	Y	Y	Y	45	3	224	13.4	**10.8 (3.45, 33.8)**	**4.90 (1.53, 15.7)**

^*^per 1000 person-years; CI, confidence interval; PCOS, polycystic ovary syndrome The adjusted hazard ratios were measured using Cox Model after controlling for age, diabetes, hypertension, hyperlipidemia, atrial fibrillation, chronic kidney disease and atherosclerosis.

Among comorbidities, diabetes alone was associated with an incident CAD of 10.5 per 1000 person-years (Table 4). The incident CAD increased to 35.2 per 1000 person-years for PSCO patients with baseline comorbidity of diabetes, with an adjusted HR of 31.0 (95% CI = 9.91–97.0) compared with women with neither PCOS nor comorbidities of diabetes, hypertension and hyperlipidemia. The corresponding adjusted HRs of CAD were 3.53 (95% CI = 1.56–7.99) for PCOS patients with baseline comorbidity of hypertension and 3.62 (95% CI = 1.97–6.66) for those with hyperlipidemia.

**Table 4:** Incidence and hazard ratio of coronary artery disease associated with polycystic ovary syndrome, diabetes, hypertension and hyperlipidemia

PCOS	DM	HTN	HL	*N*	Event	Person-years	Incidence^*^	Hazard ratio (95% CI)	
								Crude	Adjusted
N	N	N	N	31023	210	181806	1.16	1 (reference)	1 (reference)
N	Y	N	N	52	3	287	10.5	**9.04 (2.89, 28.3)**	**6.59 (2.11, 20.7)**
N	N	Y	N	347	19	1854	10.3	**9.03 (5.65, 14.4)**	**4.36 (2.68, 7.08)**
N	N	N	Y	625	14	3368	4.16	**3.70 (2.16, 6.36)**	**2.40 (1.39, 4.14)**
N	Y	Y	N	13	2	72	27.8	**23.2 (5.77, 93.5)**	**12.7 (3.14, 51.2)**
N	N	Y	Y	98	5	515	9.71	**8.86 (3.65, 21.5)**	**3.16 (1.28, 7.78)**
N	Y	N	Y	22	1	123	8.13	**7.24 (1.01, 51.6)**	6.80 (0.94, 49.0)
N	Y	Y	Y	12	5	44	113.6	**117 (48.3, 286)**	58.8 (24.1, 144)
Y	N	N	N	7432	76	44032	1.73	**1.49 (1.14, 1.93)**	**1.52 (1.17, 1.97)**
Y	Y	N	N	16	3	85	35.2	**32.8 (10.5, 102)**	**31.0 (9.91, 97.0)**
Y	N	Y	N	135	6	822	7.30	**6.26 (2.78, 14.1)**	**3.53 (1.56, 7.99)**
Y	N	N	Y	347	11	1934	5.69	**5.09 (2.77, 9.33)**	**3.62 (1.97, 6.66)**
Y	Y	Y	N	3	0	28	0	-	-
Y	N	Y	Y	83	8	419	19.1	**18.4 (9.06, 37.3)**	**8.99 (4.37, 18.5)**
Y	Y	N	Y	16	1	92	10.9	**10.2 (1.43, 72.8)**	**5.57 (0.72, 43.0)**
Y	Y	Y	Y	16	2	83	24.0	**21.2 (5.27, 85.4)**	**9.93 (2.32, 42.5)**

^*^per 1000 person-years; CI, confidence interval; PCOS, polycystic ovary syndrome The adjusted hazard ratios were measured using Cox Model after controlling for age, diabetes, hypertension, hyperlipidemia, atrial fibrillation, chronic kidney disease and atherosclerosis.

## DISCUSSION

This retrospective cohort study was conducted based on a large size of young women with PCOS at an average age of 28 years. Young female population are generally at low risk of CAD. Yet, the present study revealed that the young women with PCOS had a 63% higher incident CAD than controls without the disorder after a mean 5.9 years of follow-up. It is important to note that the excess risk of CAD did not appear during the first 2 years of follow up, reflecting that it takes at least two years to observe the significant impact of PCOS. Our data demonstrated that a single factor of disease entity such as obesity, irregular menstruation, or infertility alone was not associated with CAD risk. On the other hand, the comorbidity of diabetes, hypertension or hyperlipidemia was also associated with CAD risk and could interact with PCOS patients to increase the CAD risk further.

### Findings in relation to previous studies

Our findings can explain why patients with PCOS and patients with cardiovascular diseases share similar cardiometabolic characteristics [14–20]. The unfavorable cardiometabolic profile may present before and after the diagnosis of PCOS [8, 18, 23–29]. An UK longitudinal primary-care practices study shows that women with PCOS are 3-fold more likely to develop diabetes than controls [23]. A recent systemic study summarized findings in 35 studies that PCOS women had an adjusted odds ratio of 4.00 (95% CI 1.97–8.10) to develop diabetes and an adjusted odds ratio of 2.20 (95% CI 1.36–3.56) to develop metabolic syndrome, compared with non-PCOS controls [18]. Patients with cardiometabolic disorders are supposed at a higher risk of developing cardiovascular diseases. In our study, the prevalence of cardiometabolic comorbidities at baseline was higher in the PCOS cohort than in the control cohort. Further data analysis showed that women with the life time of cardiometabolic comorbidities had the CAD risk increased further and greater in PCOS patients than in controls (data not shown).

Previous studies on the relations between PCOS and CAD morbidity and mortality remain debatable. In the large young nurse prospective study, Solomon et al. found menstrual irregularities is associated with an adjusted HR of 1.67 for coronary heart disease after a 14-year follow-up period [21]. Menstrual irregularity is one of characteristics of PCOS. Glueck et al. even found that adolescents with oligomenorrhea are at an increased risk of metabolic syndrome and cardiovascular risk factors in later life [30]. However, two prospective studies failed to show an increased risk of CAD morbidity or CAD associated mortality for PCOS women [31, 32]. Both studies were conducted using small numbers of women with PCOS, 319 and 786 women. One retrospective study also showed no increasing risk of CAD, cancer, or death in the PCOS women after controlling for body mass index [23].

In a meta-analysis, Zhao et al. found PCOS women were at an odds ratio of 1.3 for coronary heart diseases [33]. This finding consists with our finding. We found an adjusted HR of 1.44 for developing CAD after controlling for age and cardiometabolic comorbidities. Our further data analysis showed that the adjusted HR of CAD for PCOS women declined slightly to 1.35 after controlling for age and lifetime cardiometabolic comorbidities (data not shown). Each of cardiometabolic comorbidities alone was a stronger risk factor than PCOS alone in association with the CAD development. The incidence of CAD in PCOS women comorbid with diabetes increased 20-fold, with an adjusted HR of 31. The strong impact of diabetes on PCOS patients has not been documented in previous studies. Our data also showed that women comorbid with diabetes, hypertension and hyperlipidemia simultaneously, but without PCOS, had an incident CAD of 113.6 per 1000 person-years, which was 5.4-fold greater than the incident CAD for PCOS patients with these three cardiometabolic comorbidities. Further data analysis revealed that PCOS patients comorbid with these three metabolism symptoms were at a greater risk of death than women without PCOS (3/16 vs. 0/12, or 18.8% vs. 0%). Therefore, these PCOS patients are less likely to develop CAD because of deaths.

### Strengths and limitations

The strength of our study is using a large database to evaluate the CAD risk for young Asian women with PCOS after being followed for a maximum of 16 years. The health care information of one million people was extracted from 23 million insured people in Taiwan with the ethnicity generalizable [34]. The multivariable analysis adjusting for comorbidities such as diabetes, hypertension and hyperlipidemia was to reduce the confounding effects and to increase the precision of HR measured. Accurate diagnoses of diseases can be ascertained by strict regulations of Taiwan National Health Insurance Database. We also validated the diagnoses of PCOS and CAD based on the outpatient visit frequency and inpatient diagnosis.

There are limitations in this study. Although we have controlled for the major cardiovascular risk factors, information on some risk factors was unavailable in the registry such as body mass index and detailed smoking habits. Less than 5% of women are smokers and obesity is not prevalent in Taiwan [35]. Clarifying both the dependent and independent variables using ICD9 codes was another limitation. However, previous study has shown the using ICD9 codes a good mechanism to identify disease of interest [36].

## CONCLUSIONS

In conclusion, incident CAD is greater in young women with PCOS than women without PCOS (2.25 vs. 1.38 per 1000 person-years). The incident CAD may increase further to 24.0 per 1000 person-years for PCOS patients with comorbidities of metabolism symptoms of diabetes, hypertension and hyperlipidemia. Our findings highlight that preventive interventions should be provided to women with PCOS and cardiometabolic comorbidities to reduce the risk of developing cardiovascular disease.
